# Sitting Acquisition and Early Communication Development: Are There Associations in Very Preterm Infants at Six Months of Corrected Age?

**DOI:** 10.3390/children11121538

**Published:** 2024-12-18

**Authors:** Valentina Graziosi, Chiara Suttora, Cecilia Gorini, Mariagrazia Zuccarini, Caterina Verganti, Arianna Aceti, Isadora Beghetti, Luigi Corvaglia, Annalisa Guarini, Alessandra Sansavini

**Affiliations:** 1 Department of Psychology “Renzo Canestrari”, University of Bologna, 40127 Bologna, Italy; valentina.graziosi2@unibo.it (V.G.); chiara.suttora@unibo.it (C.S.); gorini.cecilia@gmail.com (C.G.); caterina.verganti2@unibo.it (C.V.); annalisa.guarini@unibo.it (A.G.); 2 Department of Education Studies “Giovanni Maria Bertin”, University of Bologna, 40126 Bologna, Italy; mariagrazia.zuccarini@unibo.it (M.Z.); 3 Neonatal Intensive Care Unit, IRCCS Azienda Ospedaliero-Universitaria di Bologna, 40138 Bologna, Italy; arianna.aceti2@unibo.it (A.A.); isadora.beghetti@unibo.it (I.B.); luigi.corvaglia@unibo.it (L.C.); 4 Department of Medical and Surgical Sciences, University of Bologna, 40138 Bologna, Italy

**Keywords:** early motor development, early communication development, very preterm infants, sitting posture, early vocal production

## Abstract

*Background*: Research in typically and some atypically developing populations showed associations between early motor and communication development, documenting how postural development can support communicative advancements. However, these relations have scarcely been investigated in the preterm population. *Aims*: The present study aimed to describe motor (gross and fine motor) and communication (receptive and expressive) skills of very preterm infants at six months of corrected age and their associations, focusing on sitting posture achievement and early vocal production. *Methods*: Seventy very preterm infants (≤32 weeks) with no major brain injuries were assessed with the BSID-III for gross and fine motor skills, receptive and expressive language skills, and cognitive skills and were categorized as mastering (sitters), partially mastering (emerging sitters), or not mastering (non sitters) unsupported sitting. The proportional duration of sitting postures (caregiver supported, arms supported, and unsupported) in an observative section was coded with the Interact software (version 20.8.3.0). Frequency per minute of vocal utterances (vocalizations, babbling, and total) during a parent–infant play interaction was coded with the CHILDES software v11. *Results*: Correlational analyses showed significant positive associations between motor composite score and language scores (composite and expressive scaled) and between gross motor and expressive language scaled scores but a negative association between arms supported sitting duration and vocal utterances. In addition, ANCOVAs showed that sitters had significantly higher BSID-III expressive language scaled scores and vocal utterances than non sitters and emerging sitters. *Conclusions*: These findings brought new evidence linking early motor and vocal development in very preterm infants, emphasizing the importance of using observational tools alongside standardized ones to identify developmental delays and plan tailored intervention programmes.

## 1. Introduction

### 1.1. Associations Between Early Motor and Communication Skills in Typically, At-Risk, and Atypically Developing Infants

In recent decades, an increasing focus has been placed on investigating the associations between early motor and communication development, spurred by heightened interest from different theoretical perspectives. According to neuroconstructivism and dynamic systems theories [1,2], the emergence and consolidation of new motor skills in early infancy may impact communicative-linguistic development [3,4,5]. This perspective aligns with the developmental cascade hypothesis [4,6] which posits that changes in one domain (e.g., motor skills) can affect seemingly unrelated domains. Hence, attaining new motor skills can be considered a pivotal force driving comprehensive developmental processes [7]. Accordingly, mastering a novel motor skill can grant infants access to new learning opportunities, facilitating the acquisition of new skills across several developmental domains.

For instance, the acquisition of unsupported sitting, around the halfway point of the first year of life, appears to impact several domains. It enhances infants’ object exploration by increasing object accessibility and providing a more advantageous posture for manipulation [8]. Also, anatomically, the unsupported sitting posture enables opening and closing movements of the jaw with gravity, the falling forwards of the tongue, and a more readily expansion of the rib cage during respiration, which permit the production of sounds with longer durations, such as consonant-vowel units characterizing reduplicative babbling (e.g., [*babababa*] [4,9]). These assumptions suggest that the transition to unsupported sitting can mark a point for communication and language development in both typically developing [10] and atypically developing infants (e.g., children with autism spectrum disorder [11]) as well as at-risk infants (e.g., siblings of children with autism spectrum disorder [9,12]) during the first year of life.

As for typically developing infants, Libertus and Violi [10] provided evidence for motor-language associations by reporting that the emergence and development of the unsupported sitting posture from 3 to 5 months of age resulted in a strong predictor of language receptive skills assessed at 10 and 14 months of age. Other evidence comes from the study of Yingling [13] who documented significant associations between the onset of unsupported sitting and advances in consonant–vowel vocalizations in the first year of life.

Concerning at-risk infants, the literature focused on infants with an older sibling with autism spectrum disorder who often show delays in developing fundamental motor and communicative skills, regardless of whether they received the diagnosis [12]. Recent evidence suggests a link between delays in gross motor skills and communication development in infants with an older sibling diagnosed with autism spectrum disorder showing that those presenting with gross motor delays at 3 and 6 months were more likely to experience receptive and expressive language delays at 18 months. Concerning the sitting posture, Leezenbaum [14] observed a higher rate of syllabic vocalizations in 6-month-old sitters compared to non sitters among at-risk infants having an older sibling with autism spectrum disorder but not later diagnosed with developmental delays. Despite this evidence, the associations between early motor and communication development have rarely been investigated in other at-risk populations such as the preterm population.

### 1.2. Early Motor and Communication Development in Infants Born Preterm

Infants born preterm, i.e., before completing 37 weeks of gestational age, even in the absence of major brain injury, are at risk for neurodevelopmental impairments in multiple domains, such as the motor, cognitive, and communicative-linguistic domains, with more developmental disadvantages characterizing infants born extremely preterm (i.e., gestational age < 28 weeks) or very preterm (i.e., gestational age < 32 weeks) than those born moderately preterm (i.e., gestational age of 32–33 weeks) and late preterm (i.e., gestational age of 34–36 weeks) [15,16,17,18,19].

Regarding early motor development, preterm infants exhibit delayed gross motor abilities and motor milestone achievements in the first year’s corrected age compared to full-term peers [20,21]. Notably, a systematic review by Boonzaaijer et al. [22] provided an overview of factors associated with early gross motor development of healthy full-term and preterm infants with a gestational age ranging from 26 to 42 weeks, finding moderate evidence that a shorter gestational age was associated with a delay in gross motor development in the first 18 months of life and strong evidence that very low birth weight (<1500 g) and low birth weight (1500–2500 g) were associated with poorer motor outcomes from 0 to 24 months. Only in the last few years, the literature began to more deeply address the motor developmental profiles of very preterm infants without severe perinatal complications, highlighting a poorer trunk and postural control in preterm infants compared to full-term peers in the first year of corrected age [23] and high interindividual variability in motor developmental profiles among preterm infants in the first two years corrected age [24].

Regarding posture acquisition, Valentini and colleagues [25] revealed that preterm infants, cross-sectionally assessed with the Alberta Infant Motor Scale [26] in the 0–12 month corrected age range, had lower scores in more control-demanding postures than full-term peers. The study showed different periods of intensive motor learning for sitting acquisition that spanned from 1 to 7 months of life for full-term infants and from 3 to 7 months for preterm infants. Further evidence comes from the study of Pin et al. [27] reporting that only 56% of the very and extremely preterm infants observed at 8 months of corrected age could maintain sitting very briefly without arm support (emerging unsupported sitting), compared to 90% of their full-term peers. Delays found in motor acquisition and consolidation in preterm infants concerned not only gross motor and postural skills but also fine motor skills, as revealed by a few studies. Soares et al. [28] reported that infants born late preterm demonstrated less mouthing than full-term peers from 5 to 7 months of age. Accordingly, Lobo et al. [29] found that extremely preterm and very preterm infants spent less time exploring objects throughout the first 6 months of corrected age than did full-term peers, showing less variability in individual behaviours. Furthermore, Zuccarini et al. [30,31] highlighted less manual exploration at 6 months of corrected age and different oral and manual object exploration patterns from 6 to 9 months of corrected age in extremely preterm infants compared to full-term peers. The motor domain appears thus to be negatively impacted by neonatal immaturity from the first year of life.

With regard to the communicative-linguistic domain, delays have been reported in extremely and very preterm infants, with some difficulties also observed in moderately preterm infants, from 1 to 5 years of age [16,17,32], often associated with delays in other developmental domains [33]. However, limited knowledge is available about the impact of preterm birth on the development of vocalizations and babbling during the first year of life. Regarding early vocal production, preterm infants were reported to produce significantly fewer vocalizations and prelinguistic sounds than full-term peers at 6 months of corrected age [34]. In line with these findings, Nardelli de Oliveira et al. [35] found delayed babbling onset in late preterm-born infants around 9 months of age compared to full-term peers, and Strandberg et al. [36] showed significantly less canonical babbling and a restricted consonant inventory in extremely preterm infants relative to full-term peers at 12 months of corrected age. Furthermore, only one-third of extremely preterm infants produced their first words by the corrected age of 12 months [37], suggesting a persistent communicative-linguistic delay during the first year of life. To conclude, very and extremely preterm infants appear particularly vulnerable in the domain of communication and language from the early stages of development.

### 1.3. Associations Between Early Motor and Communication Development in Infants Born Preterm

Research on the associations between early motor and communication development in preterm infants is scarce. Recently, Jensen-Willet et al. [38] assessed very preterm infants with the Bayley Scales of Infants and Toddler Development (BSID-III [39]) at 6 months of corrected age. In this study, unsupported sitting was assessed with the BSID-III motor screening item (item #11), classifying infants as sitters if they sat for more than 60 s hands free. Among participants, 31% were sitters and 69% were non sitters. BSID-III cognitive and language composite scores were significantly higher in very preterm sitters than non sitters, who also differed in their neonatal immaturity as they were born earlier with lower birth weights and required respiratory support for a longer period [38]. Specifically, a reduction in ventilation days, along with increased seated time, explained 16.8% of the total variance in BSID-III language scores, underlining the impact of medical risks on language development in these infants. The above findings suggest that achieving a proper sitting posture is associated with more advanced communicative-linguistic skills in very preterm infants and that preterm birth and the clinical risk factors associated with it impact sitting achievement. However, it remains unclear whether sitting achievement is related with both receptive and expressive language development in very preterm infants at 6 months of corrected age. Associations were also found between early motor and communicative-linguistic skills in extremely preterm infants. Indeed, Zuccarini et al. [40] showed that manual object exploration at 6 months of corrected age was predictive of both gesture and vocal production at 12 months of corrected age. In addition, Benassi et al. [37] found significant associations between fine motor skills and representational gesture production in extremely preterm infants at 12 months of corrected age. However, to our knowledge, no studies have explored the associations between fine motor and communicative-linguistic skills in very preterm infants at 6 months of age.

### 1.4. The Current Study

The present study, by employing both standardized and observational tools, aimed to describe motor (gross and fine motor) and communication (receptive and expressive) skills of very preterm infants at 6 months of corrected age. We decided to use corrected age to account for infants’ level of neuropsychological maturation, a common practice in studies conducted during the first two years of life [16,17]. Because full-term pregnancy is defined as 40 weeks of gestation [41], corrected age was calculated by subtracting the number of weeks a child was born prematurely from her/his chronological age [42].

The present study also aimed to investigate the association between motor and communication skills, with a focus on sitting posture achievement and early vocal production controlling for the infants’ corrected age and cognitive development. We decided to control for these variables to take into account interindividual variability. This approach is particularly important given that cognitive abilities in children are known to be associated with both motor and language development within the first two years of life [43,44].

We expected that very preterm infants would exhibit a slower development of gross motor, fine motor, receptive, and expressive skills at 6 months of corrected age, compared to normative values. We also expected to observe an association between motor and communication skills and, particularly, between gross motor and expressive skills, and that very preterm infants mastering unsupported sitting at 6 months of corrected age would show higher expressive language scores and more vocal productions than very preterm infants partially mastering or not mastering unsupported sitting. Regarding the relation between fine motor and communication (receptive and expressive) skills, the study had an explorative aim.

## 2. Materials and Methods

### 2.1. Participants

The present study included 70 very preterm infants (33 females, 37 males) (see Table 1). All infants were born at the Policlinico di Sant’Orsola Hospital of the IRCCS Azienda Ospedaliero-Universitaria di Bologna, Italy, and after their discharge, they were enrolled in the hospital preterm follow-up programme. Within this programme, infants were invited to participate in the study if they had: (a) a gestational age ≤ 32 weeks; and (b) no major brain injuries (i.e., grade III intraventricular haemorrage, periventricular haemorragic infarction, periventricular leukomalacia or diffuse white matter injury, post-haemorragic ventricular dilatation), nor congenital malformations, visual (i.e., blindness, severe retinopathy of prematurity) or auditory (i.e., deafness) disabilities. Clinical data were collected from infants’ medical records, whereas family socio-demographic data were gathered from parents through a questionnaire and an interview.

The study received formal approval from the local Research Ethical Committee Comitato Etico di Area Vasta Emilia Centro (approval numbers: 76/2013/U/Sper/AOUBo; EM 194–2017; EM 193–2018; EM 1229–2020). The parents of all infants gave written informed consent for participation in the study, data analysis, and anonymized data publication.

### 2.2. Procedure

Very preterm infants, along with their parents, were invited to participate in an assessment session at approximately 6 months of corrected age (*M* = 6.15 months, *SD* = 0.38, range = 5.35–7.15; chronological age: *M* = 8.63, *SD* = 0.71, range = 7.48–10.40). The assessment was conducted in a quiet room of the Neonatology Unit of the Policlinico di Sant’Orsola Hospital, equipped with a video recording system, a wall mirror, a spacious mat, and an infant highchair. The total duration of the assessment averaged approximately one hour.

### 2.3. Tools

#### 2.3.1. Standardized Assessment of Motor, Language, and Cognitive Skills

Motor, language, and cognitive skills were assessed with the Italian version [50] of the Bayley Scales of Infant and Toddler Development, Third Edition—BSID-III [39]. The BSID-III [39] provides norm-referenced standardized composite motor, language, and cognitive scores (*M* = 100, *SD* = 15), and standardized scaled scores (*M* = 10, *SD* = 3), respectively, for the gross motor and fine motor subscales and the receptive and expressive language subscales for infants/toddlers aged one to 42 months and 15 days. BSID-III standardized composite and scaled scores for our sample were calculated by referring to the normative values of the original standardization [39] as normative values for the Italian population were not available for the first year of life yet. The BSID-III is a valid, reliable, and widely used tool used for clinical and research purposes in studies on preterm and full-term infants [17]. The BSID-III was administered by two trained psychologists (the first and the fourth author) with the infant seated in a highchair with age-appropriate toys for the fine motor, language, and cognitive assessment. The gross motor assessment was conducted by positioning the infant on a spacious mat with a caregiver sitting close to the infant to provide comfort when required. In particular, unsupported sitting was assessed with the Italian BSID-III gross motor subscale items (items #26 and #22), respectively, classifying infants as: maintaining the unsupported sitting posture for 30 or more consecutive seconds, which we defined as “sitters” (item #26); maintaining the unsupported sitting posture for 5 or more consecutive seconds (but less than 30 s), that we defined as “emerging sitters” (item #22); we defined “non sitters” as those infants maintaining the unsupported sitting posture for less than 5 consecutive seconds or not maintaining it yet. The BSID-III assessment lasted about 30 min.

#### 2.3.2. Sitting Posture Observation Session and Coding

The sitting posture was video recorded on the mat where the gross motor assessment took place. During the observation, which lasted, on average, 115 s (*SD* = 96), the caregiver was instructed to place the infant on the mat, starting from the infant’s preferred posture to help him/her adjust to the new environment. After a few minutes, the examiner requested the caregiver to move the infant to the sitting posture like they would do at home. When an infant did not spontaneously exhibit the sitting posture, the examiner or caregiver provided support through positioning or physical prompting, according to the BSID-III item #22 instructions: “With the child seated, provide pelvic support by placing your hands around the child’s lower back. According to the child’s ability to sit alone, gradually loosen your hold”.

The sitting posture was coded by a trained coder (the first author) with the Interact software (version 20.8.3.0) [51]. This software is a time-linked, computer-based video interface commonly used in research to analyze the progression of behaviours and emotions during interactions between children and between adults and children [52,53].

The following coding scheme was developed by considering the literature on this topic [26,54] to allow for a detailed frame-by-frame analysis of each infant’s time spent in three types of sitting postures: (a) Caregiver supported: the sitting position is maintained only when the infant is supported by the caregiver or the examiner; (b) Arms supported: the infant can maintain the sitting posture supporting his/her weight on propped arms; (c) Unsupported: the sitting posture is maintained without propped arms or external support so that the infant can sit independently. Sitting posture categories were considered mutually exclusive, and episodes were coded if they lasted at least one second using an event-based coding procedure. Coding started when infants were placed on the mat to observe the sitting posture and ended when infants stopped performing sitting positions. Proportional duration for each type of sitting was calculated over the total time of observed sitting.

#### 2.3.3. Vocal Production Observation Session and Coding

Infants’ spontaneous vocal production was video recorded during a semi-structured parent–infant play interaction. The parent sat on a chair in front of their infant, seated in a highchair. They were asked to play with their infant as they did at home with a set of age-appropriate toys (i.e., rattles, toys for teething, and colourful toys) for an average duration of seven minutes (*M* = 7.23, *SD* = 1.72). A trained observer (the third author) transcribed and coded the identified vocal productions with the CHAT of CHILDES software v11 [55]. Infants’ vocal productions were identified based on the following criteria according to previous studies [37,56,57]: the vocal utterance occurred on a single egress of the breathing cycle; it was judged to be non-meaningful and speech-like; it included at least one voiced vocalic element or a voiced syllabic consonant. Vocal utterances were coded as separate when they were bounded by 1 s of silence on either side, a breath, adult speech, or falling intonation ([54], adapted). Vocal utterances were classified into one of two mutually exclusive categories according to the following coding scheme, adapted from previous studies [37,56,57,58,59]: vocalizations (full vowels, quasivowels, squeals and growls, whispers and yells, ingressive sounds, raspberries, clicks, goos, glottal stop sequences, consonants, semiconsonants, consonant-vowel or vowel-consonant sequences in which the consonant was a glide or glottal); babbling (canonical or reduplicated sequences of consonant-vowel or vowel-consonant in which the place and manner of articulation remain constant). Reflexive sounds (i.e., cry, laugh, and vegetative sounds) were not analyzed for the aims of the present study. The frequency per minute of vocal utterances (vocalizations, babbling, and total vocal utterances given by the sum of vocalizations and babbling) was computed with the CLAN of CHILDES software.

### 2.4. Reliability

Inter-rater reliability for the coding of infants’ sitting postures was assessed with a two-step double-blind procedure for 23% of the sessions (16 infants), coded by a second independent trained observer with the Mangold Interact software. In the first step, the percentage agreement of infant sitting postures detected by the two independent observers was computed: the percentage agreement obtained was 82%. Secondly, Cohen’s kappa [60] was calculated on the distinction among sitting postures (caregiver supported, arms supported, and unsupported), with a result of 0.92.

Inter-rater reliability for coding infants’ vocal productions was assessed in 20% of the sessions (14 parent-infant interactions) using the same two-step double-blind procedure. The percentage agreement between the two independent observers in detecting infant vocal utterances was 87%. Cohen’s kappa, calculated to distinguish between vocalizations and babbling utterances, revealed no discrepancies between observers, resulting in a K value of 1.

### 2.5. Plan of Analyses

Data analyses were performed with IBM SPSS Statistics 27 (Armonk, NY, USA), using a bilateral test with *p* set at <0.05. Data were tested for normality with the Kolmogorov–Smirnov test and skewness and kurtosis values, considering normal data ranging between −2 and 2. As sitting postures’ proportional durations (caregiver supported, arms supported, and unsupported) and vocal productions’ frequency per minute (vocalizations, babbling, and total vocal utterances) were not normally distributed, they were transformed in rank to use parametric analyses.

Descriptive analyses were performed on BSID-III composite (motor, language, cognitive) and scaled (gross motor, fine motor, receptive, and expressive) scores, sitting (caregiver supported, arms supported, and unsupported) postures’ proportional durations, and vocal utterances’ (vocalizations, babbling, and total) frequency per minute.

Partial correlational analyses, partialized for corrected age and BSID-III cognitive score, looked at the associations between infants’ motor and communication skills. The choice of controlling for corrected age and BSID-III cognitive score was made to take into account interindividual variability, as infants ranged from 5.35 to 7.15 months for corrected age and from 80 to 130 for the cognitive score.

A first set of Pearson partial correlational analyses was carried out to assess the associations between BSID-III motor scores (composite, gross motor, and fine motor), BSID-III language scores (composite, receptive, and expressive), and vocal utterances’ frequency per minute (vocalizations, babbling, and total).

A second set of Pearson partial correlational analyses was conducted to assess the associations between sitting postures’ proportional duration (caregiver supported, arms supported, unsupported), BSID-III language scores (composite, receptive, and expressive), and vocal utterances’ frequency per minute (vocalizations, babbling, and total).

To examine whether non sitters, emerging sitters, and sitters significantly differed in BSID-III language scores (composite, receptive, and expressive) and the frequency per minute of vocal utterances (vocalizations, babbling, and total), a set of ANCOVAs was planned. These analyses aimed to control for corrected age and the BSID-III cognitive score as covariates. To determine whether these covariates should be included in the ANCOVAs, firstly, two one-way ANOVAs were conducted to verify whether the covariates (corrected age and Bayley Cognitive Composite score) differed across the three groups: sitters, non sitters, and emerging sitters. The three groups significantly differed in the corrected age, *F*(2,67) = 9.20; *p* < 0.001; *η_p_*^2^ = 0.22, with sitters having a higher corrected age compared to both emerging sitters and non sitters (*ps* < 0.005). By contrast, the three groups did not significantly differ in the Bayley Cognitive Composite Score, *F*(2,67) = 1.30; *p* = 0.28; *η_p_*^2^ = 0.04. As a result, only corrected age was included as a covariate in the ANCOVAs.

For each ANCOVA, we first tested the assumption of homogeneity of regression slopes by including an interaction term between the covariate and the independent variable. The assumption of homogeneity of regression slopes was satisfied, as no significant interactions were found between corrected age and the dependent variables (BSID-III language scores and vocal utterances). We thus proceeded to run the ANCOVAs without the interaction term.

## 3. Results

### 3.1. Descriptive Analyses

Regarding BSID-III motor scores (see Table 2), the composite score and the gross motor scaled score fell within the low normal range, whereas the fine motor scaled score was closer to the mean normal range. Regarding BSID-III language scores, the composite score, the receptive, and the expressive scaled scores fell within the low normal range (see Table 2). Regarding the BSID-III cognitive composite score, the mean value fell within the mean normal range (see Table 2).

Looking at interindividual differences in sitting posture achievement, 75.7% (*n* = 53) of the infants were non sitters, 12.8% (*n* = 9) emerging sitters, and 11.4% (*n* = 8) sitters.

Regarding observational measures, proportional durations of sitting postures (caregiver supported, arms supported, and unsupported) and frequency per minute of vocal utterances (vocalizations, babbling, and total) are reported in Table 3. As for sitting postures, infants spent most of their time (70%) sitting with the support of their caregivers compared to arms supported (17%) and unsupported sitting (13%) (see Table 3). Concerning vocal productions, infants produced mainly vocalizations with respect to babbling, the latter still infrequent (see Table 3). Indeed, 95.5% (*n* = 64) of the infants produced vocalizations, whereas only 25.7% (*n* =18) of the infants produced babbling.

### 3.2. Associations Between Motor and Communication Skills

Associations between BSID-III motor scores (composite, gross motor scaled, and fine motor scaled), BSID-III language scores (composite, receptive scaled, and expressive scaled), and vocal utterances’ (vocalizations, babbling, and total) frequency per minute are reported in Table 4. Positive associations emerged between BSID-III motor composite score and BSID-III language composite and expressive scaled scores. Positive associations were also found between the BSID-III gross motor scaled score and the expressive language scaled score (see Table 4). These significant correlations showed an effect size comprised between small to medium.

Associations between sitting postures (caregiver supported, arms supported, and unsupported) proportional durations and vocal utterances’ (vocalizations, babbling, and total) frequency per minute, and BSID-III language scores (composite, receptive scaled, and expressive scaled) are reported in Table 5. Negative associations were found between time spent by infants in arms supported sitting posture and vocal utterances’ frequency per minute both for vocalizations and total vocal utterances with an effect size comprised between small to medium. No other significant associations were found.

### 3.3. Differences Among Non Sitters, Emerging Sitters, and Sitters in Communication Development

**Figure 1:**
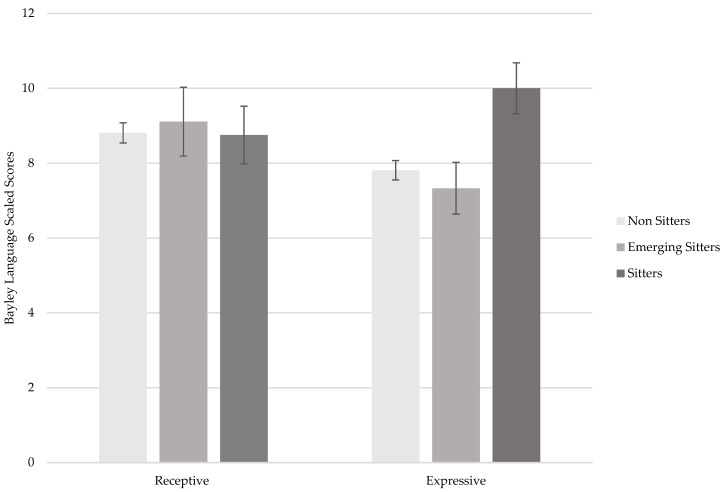
BSID-III receptive and expressive language scaled scores in non sitters, emerging sitters, and sitters. Note. Error bars represent standard errors.

Results from the ANCOVAs showed that sitters had significantly higher scores than non sitters and emerging sitters (*ps* < 0.005) in the BSID-III Expressive scaled score, *F*(2,66) = 4.45; *p* = 0.015; *η_p_*^2^ = 0.12 (see Table 6 and Figure 1). No significant group differences were found for the BSID-III language composite score, *F*(2,66) = 1.51; *p* = 0.23; *η_p_*^2^ = 0.04) and the Receptive scaled score, *F*(2,66) = 0.11; *p* = 0.89; *η_p_*^2^ = 0.01, as shown in Table 6 and Figure 1. The covariate (corrected age) did not have a significant effect in these models.

**Figure 2:**
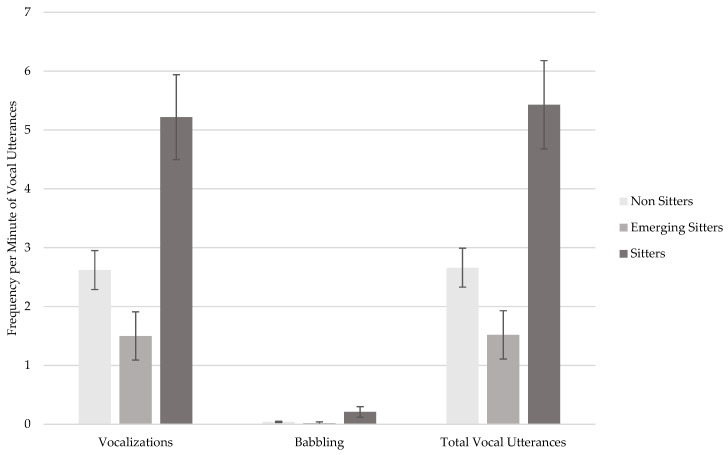
Frequency per minute of vocalizations, babbling, and total vocal utterances in non sitters, emerging sitters, and sitters. Note. Error bars represent standard errors.

Results from the ANCOVAs also revealed that sitters produced a significantly higher frequency per minute of vocalizations, *F*(2,66) = 6.51; *p* = 0.003; *η_p_*^2^ = 0.17, and total vocal utterances, *F*(2,66) = 6.61; *p* = 0.002; *η_p_*^2^ = 0.17, than non sitters and emerging sitters (*ps* < 0.001; see Table 6 and Figure 2). By contrast, there were no significant group differences in the frequency per minute of babbling, *F*(2,66) = 1.34; *p* = 0.27; *η_p_*^2^ = 0.04, as shown in Table 6 and Figure 2. The corrected age showed no significant effect as covariate on these outcomes.

## 4. Discussion

Our findings broaden the knowledge of motor and communication skills and profiles of very preterm infants at 6 months of corrected age, providing new insights into the growing literature on motor–language associations. Significant evidence of positive associations between motor and, particularly, gross motor, and communication development was found with higher expressive language scores and more vocal productions characterizing infants mastering unsupported sitting. Using both standardized and observational measures allowed us to collect information at multiple levels of analysis, offering more details on motor and communication skills and new insight for future research and clinical practice.

### 4.1. Motor and Communication Skills of Very Preterm Infants at 6 Months

Our findings showed that mean BSID-III motor and language scores of very preterm infants at 6 months of corrected age fell within the low normal range compared to the normative values of the original standardization [39], highlighting large interindividual variability and the existence of some vulnerabilities in this population. With regard to the motor domain of our sample specifically, the gross motor functional area appeared weak, whereas the fine motor function was apparently more preserved. Also, concerning the communication domain, the expressive function appeared weak, whereas the receptive function was seemingly more preserved. These findings align with those of previous research, which also found lower motor skills, particularly gross motor ones [61,62], and poorer communication skills, particularly expressive skills, in very preterm infants compared to their term-born peers in the first year of life [36,37].

Behavioural observation provided further insights into very preterm infants’ postural skills, validating our expectations. As for sitting postures, on average our sample spent most of the time sitting with the support of the caregiver, whereas very limited time sitting with the support of own arms or unsupported. Our findings confirmed and expanded upon previous research on postural development in the preterm population that highlighted, compared to full-term peers, poorer postural control in extremely and very preterm infants during the first months [63,64] and in the second half of the first year’s corrected age [23,65]. Delayed segmental trunk control and sitting posture were indeed observed in moderately preterm infants at 6–7 months of corrected age [66] and in extremely preterm infants at 8 months of corrected age [27]. These delayed postural achievements in preterm infants may be explained by muscle power regulation difficulties and hyperextension of the trunk characterizing this population in the first year of life [67,68].

Our results also offered further insights into interindividual differences in unsupported sitting achievement by very preterm infants at 6 months of corrected age. Indeed, in our sample, most infants (75.7%) were non sitters (maintaining unsupported sitting for less than 5 consecutive seconds or not maintaining it yet), whereas only a few were emerging sitters (12.8%, maintaining unsupported sitting for 5 or more consecutive seconds but less than 30) or sitters (11.4%, maintaining unsupported sitting for 30 or more consecutive seconds). Our findings thus highlighted that most very preterm infants have not mastered unsupported sitting at 6 months of corrected age yet, differently from typically developing full-term infants who, on average, show unsupported sitting or propped arm sitting, with the torso supported by the hands and arms, around 6–7 months of age [4]. Our findings brought further evidence to two previous studies highlighting a slow achievement of unsupported sitting in the preterm population, finding it only in 31% of a very preterm sample at 6 months of corrected age [38] and 56% of an extremely preterm sample, compared to 90% of a full-term sample, at 8 months of corrected age [27]. As regards early motor developmental profiles, Suir et al. [24], using cluster analysis, highlighted three motor developmental profiles (i.e., early developers, gradual developers, and late bloomers) in very preterm Dutch infants, assessed with the Alberta Infant Motor Scale between 5 and 17 months of corrected age. These authors showed that gradual developers and late bloomers had slower developmental curves, compared to their full-term peers, from 12 months of corrected age onwards [24]. Our findings, by identifying three groups based on sitting posture achievement, provided a new framework to address this interindividual variability, starting from 6 months of corrected age. Future research is needed to assess how effectively these early sitting profiles can predict later motor developmental outcomes in very preterm children.

Regarding early vocal production, we found that very preterm infants, at 6 months of corrected age, produced mainly vocalizations, whereas babbling was still rarely produced. Our findings brought new evidence on very preterm infants’ early vocal production, which has been scarcely investigated. In particular, we corroborated, with a larger sample, previous findings by Salerni et al. [34], who assessed very preterm infants at 6 months of corrected age, and expanded their findings, with regard to interindividual differences, showing that almost all very preterm infants of our sample produced vocalizations, whereas only one out of four produced babbling. Our findings thus showed that interindividual differences in early vocal production among very preterm infants can already be assessed at 6 months of corrected age and these differences might account for the slower vocal production observed in very and extremely preterm infants between 8 and 12 months of corrected age [36,37].

### 4.2. Associations Between Motor and Communication Skills and Different Profiles in Very Preterm Infants

Our study revealed significant associations between motor and communication skills in very preterm infants at 6 months of corrected age, as measured by standardized and observational tools. We found small to medium positive correlations between the BSID-III motor scores (including both composite and gross motor scaled scores) and the BSID-III language scores (covering both composite and expressive scaled scores). Our findings brought new evidence, with regard to the very preterm population, about the association between gross motor and expressive language skills, previously found in typically developing infants and in siblings of children with autism spectrum disorder [9,10,14]. Our findings also showed that very preterm sitters had significantly higher BSID-III expressive language scaled scores and produced more vocalizations and total vocal utterances (vocalizations plus babbling) compared to very preterm non sitters and emerging sitters. To our knowledge, this is the first study reporting the presence of significant associations between mastering unsupported sitting and vocal production in very preterm infants at 6 months of age, assessing these abilities using both standardized and observational measures. The previous literature, like the study by Jensen-Willet et al. [38], using standardized measures, reported that the BSID-III language composite score was significantly higher in preterm sitters than non sitters at 6 months of corrected age, but it did not examine whether this association pertained to both receptive and expressive abilities, it did not control for cognitive development, and it did not use observational measures beside standardized ones. Our findings thus provided new insights into the previous literature focused on the preterm population highlighting the presence of strict associations between the motor and communication domains in the absence of major brain injury, and clarifying the contribution of gross motor abilities to the expressive more than the receptive skills at 6 months of corrected age, controlling for cognitive development.

By contrast, no significant associations were found between fine motor skills and communicative-linguistic skills. Apparently, fine motor skills of very preterm infants in our sample were more preserved than gross motor skills. However, it might be possible that employing observational measures, besides standardized ones, to investigate fine motor skills, would have provided more insights into this issue. Indeed, as most of the very preterm infants in our sample did not master unsupported sitting yet, which increases object accessibility and provides a more favourable posture for manipulation [8], it is conceivable that they had limited opportunities to engage in exploratory activities and train fine motor skills, an aspect that could have more deeply been assessed using also observational measures, as previously conducted by Zuccarini et al. [31].

Another new finding of our study regards the presence of significant negative associations between the time spent by infants in arms supported sitting and their vocal productions during parent–infant play interaction. To our knowledge, this is the first study exploring how arms supported sitting posture relates to early vocal production. As mentioned above, unsupported sitting seems to provide infants with a new and expanded view of their surroundings, increasing visual access to the physical and social environment [4,10]. Conversely, as suggested by our findings, arms supported sitting, although promoting the transition from supported to unsupported sitting, seems to temporarily limit infants’ communicative behaviours. Indeed, when infants are learning to sit by supporting themselves with their own arms, they are mainly focused on maintaining a balanced posture, having thus fewer opportunities to explore their surroundings and engage in social interactions. To this regard, Berger et al. [69], examining 7-month-old typically developing infants, showed that, as long as infants had not mastered balance control in sitting, they had fewer attentional resources for engaging in other motor and cognitive behaviours. Furthermore, as their trunk is not upright and the rib cage does not expand, infants have fewer opportunities to manipulate objects and vocalize [4]. Indeed, infants who can sit while keeping their arms free explore more than infants who need to prop themselves up with their hands [70]. Furthermore, caregivers are more likely to label objects when they are being held, looked at, and manipulated by the infant [71], and object manipulation and exploration are more enhanced by unsupported sitting than by external or arms supported sitting [72]. Arms supported sitting strategy, thus, although effective for the transition from supported to unsupported sitting, may temporarily limit social and communicative opportunities.

Summing up, our findings pointed out that infants’ ability to sit without support for a proper amount of time, with the infant managing to maintain a good balance, can foster vocal productivity and, overall, more advanced expressive skills. Our findings offer thus further insights into the hypothesis that achieving good trunk control reorients the position of the speech apparatus allowing infants to produce sounds for longer periods and changes how infants interact with objects and their surroundings, reorganizing the physical space in which caregiver-infant interaction happens [4].

### 4.3. Limits and Future Directions

This work has considerable strengths including a representative sample of 6-month-old very preterm infants with no major brain injuries and a motor and communication assessment with both standardized and observational tools. However, our study faced some limitations, raising important questions for future research.

First, it was not possible to recruit a full-term comparison sample. This was due to the timing of the study, which began during the COVID-19 pandemic, representing a significant challenge to clinical research [73]. Due to sanitary and management challenges, only very preterm infants who needed a clinical follow-up, but not full-term ones, could be recruited at the University Hospital. Future studies should thus compare Italian very preterm and full-term infants regarding early motor and communication skills and their associations. This comparison would allow to consider the role of the Italian cultural context in sitting acquisition. Indeed, as regards typical development, an enormous variation in sitting proficiency within and across cultural groups was found in a cross-cultural study on 5-month-old typically developing infants belonging to six different countries (Argentina, Cameroon, Italy, Kenya, South Korea, and the United States) [74]. One-third of the sample exhibited unsupported sitting with sitting bouts of 5 or more consecutive seconds at 5 months of age; most of these infants belonged to Kenya and Cameroon, a few of them to Argentina, South Korea, and the United States and none of them to Italy [74]. The recruitment of a full-term comparison sample in future studies would also allow to collect BSID-III normative values for the Italian population that are not available for the first year of life yet. For this reason, in the current study the BSID-III standardized scores were calculated by referring to the normative values of the original standardization [39].

Second, the assessment of fine motor skills was limited to standardized measures. Further research should examine individual differences in fine motor skills and associations between manual object exploration and communicative-linguistic skills by using observational measures, besides standardized ones, in very preterm and full-term infants.

Third, although we collected clinical and socio-demographic data about infants and parents, more detailed information is needed regarding environmental characteristics and daily practices of infants’ caregivers. The literature indeed emphasizes that the presence of toys and stimulating materials in the home environment plays a crucial role in preterm infants’ motor development and, therefore, requires more attention [75,76]. Future research should address the role of the home environment, including the quality of caregiver-infant interaction and parent input, in understanding individual differences and promoting motor development in preterm infants.

In addition, future work is necessary to examine longitudinal associations, besides concurrent ones, between motor and communication skills in the preterm population during the first two years of life. A recent study by Ross et al. [77] found that, at 18 months of corrected age, motor delays were related to poorer language skills in extremely preterm infants, but future research is needed to deeply explore this association at multiple points between 6 and 24 months of corrected age with both standardized and observational measures and to examine how the achievement of main motor milestones, such as sitting and walking, could influence language development in very preterm infants.

## 5. Conclusions

The current study provided new methodological and clinical evidence to inform health professionals caring for very preterm infants, emphasizing the importance of identifying early motor and communication weaknesses, such as those regarding the achievement of unsupported sitting and babbling, and addressing them within a multidomain and multimethod approach. This approach aligns with that suggested for the achievement of later motor and communication milestones, such as walking, gestures, and words, for typically developing infants and siblings of children with ASD [5,78].

Our findings also highlighted the importance of increasing healthcare professionals’ awareness of multidomain interventions, such as those involving both motor and communication domains, that can maximize the impact on development. Recent studies [79,80] have indeed shown that early developmental interventions for preterm infants focusing on both parent–infant relationships and infant development, home-based, family-centred, and multiple domains are more likely to have a positive effect on motor, perceptual, and cognitive development over the short to medium term compared with standard care.

## Figures and Tables

**Table 1:** Clinical and socio-demographic characteristics of participants.

	Very Preterm Infants	
	(*n* = 70)	
Participants’ Characteristics	*M/n*	*SD/%*
Gestational age (weeks), *M, SD*	29.12	2.62
Birth weight ^a^ (grams), *M, SD*	1199.54	380.69
Length of stay in hospital (days), *M, SD*	66.69	35.78
Female, *n, %*	33	47.1
Firstborn, *n, %*	44	62.8
Siblings (≥1), *n, %*	43	61.4
Multiple births, *n, %*	28	40
Cesarean section, *n, %*	53	75.7
IUGR, *n, %*	7	10
RDS, *n, %*	55	78.6
Apnoea, *n, %*	10	14.3
BPD, *n, %*	16	22.9
IVH I/II, *n, %*	4	5.7
ROP I/II, *n, %*	9	12.9
Sepsis, *n, %*	9	12.9
Mechanical Ventilation (MV), *n, %*	14	20
Multiple medical complications (≥2), *n, %*	38	54.3
Mother’s age ^b^ (years), *M, SD*	34.67	6.11
Father’s age ^c^ (years), *M, SD*	37.72	7.08
Mothers with a high educational level (>13 years), *n, %*	34	48.6
Fathers with a high educational level ^d^ (>13 years), *n, %*	30	42.8
Mother’s nationality (Italian), *n, %*	49	70
Father’s nationality (Italian), *n, %*	47	67.1

Note. Medical complications (infants could have one or more medical complications): IUGR: Intrauterine Growth Restriction [45]; RDS: Respiratory Distress Syndrome [46]; BPD: Bronchopulmonary Dysplasia [47]; IVH I/II: Intra-Ventricular Hemorrhage [48]; ROP I/II: Retinopathy of Prematurity [49]. Multiple medical complications: infants with 2 or more of the above complications. Data were missing for: ^a^ 1 participant; ^b^ 4 participants; ^c^ 5 participants; ^d^ 1 participant.

**Table 2:** Descriptive statistics of the BSID-III motor, language, and cognitive scores.

	Very Preterm Infants			
	(*n* = 70)			
Measures	*M*	*SD*	*Min*	*Max*
BSID-III Scores				
Motor Composite	91.59	13.43	58	118
Gross Motor Scaled	7.50	2.49	2	16
Fine Motor Scaled	9.67	3.34	1	16
Language Composite	91.06	9.64	59	115
Receptive Scaled	8.84	2.10	3	12
Expressive Scaled	8.00	2.01	3	13
Cognitive Composite	99.29	9.68	80	130

**Table 3:** Descriptive statistics of sitting posture and vocal utterances.

	Very Preterm Infants			
	(*n* = 70)			
Measures	*M*	*SD*	*Mdn*	*IQR*
Sitting ^a^				
Caregiver supported	0.70	0.37	0.86	0.64
Arms supported	0.17	0.27	0.00	0.22
Unsupported	0.13	0.29	0.00	0.09
Vocal utterances ^b^				
Vocalizations	2.77	2.45	1.85	3.80
Babbling	0.06	0.14	0.00	0.10
Total	2.83	2.49	1.92	3.80

Note. ^a^ proportional duration; ^b^ frequency per minute.

**Table 4:** Partial Pearson correlations (partialized for corrected age and cognitive score) between BSID-III motor scores, BSID-III language scores, and vocal utterances.

Pearson Correlations	BSID-III Language Scores			Vocal Production		
	Composite	Receptive Scaled	Expressive Scaled	Vocalizations	Babbling	Total
BSID-III Motor Scores						
Composite	0.27 *	0.21	0.24 *	0.10	0.20	0.11
Gross Motor Scaled	0.23	0.14	0.23 *	0.20	0.09	0.20
Fine Motor Scaled	0.17	0.17	0.13	−0.03	0.19	−0.02

Note. * *p* < 0.05.

**Table 5:** Partial Pearson correlations (partialized for corrected age and cognitive composite score) between sitting posture, BSID-III language scores, and vocal utterances.

Pearson Correlations	BSID-III Language Scores			Vocal Utterances		
	Composite	Receptive Scaled	Expressive Scaled	Vocalizations	Babbling	Total
Sitting ^a^						
Caregiver supported	−0.09	−0.06	−0.07	0.02	0.01	0.04
Arms supported	0.01	0.04	−0.07	−0.24 *	−0.07	−0.26 *
Unsupported	0.12	0.10	0.11	−0.01	−0.03	0.01

Note. ^a^ proportional duration; * *p* < 0.05.

**Table 6:** Descriptive statistics of BSID-III language scores and vocal utterances in non sitters, emerging sitters, and sitters.

	Non Sitters		Emerging Sitters		Sitters	
	(*n* = 53)		(*n* = 9)		(*n* = 8)	
Measures	*M*	*SD*	*M*	*SD*	*M*	*SD*
BSID-III Language Scores						
Composite	90.45	8.76	89.78	13.53	96.50	9.88
Receptive Scaled	8.81	2.00	9.11	2.76	8.75	2.19
Expressive Scaled	7.81	1.87	7.33	2.06	10.00	1.92
Vocal utterances						
Vocalizations	2.62	2.44	1.50	1.23	5.22	2.05
Babbling	0.04	0.11	0.02	0.06	0.21	0.28
Total	2.66	2.46	1.52	1.23	5.43	2.13

## Data Availability

The dataset presented in this article is not readily available because it includes sensitive information about minors with developmental vulnerabilities. Requests to access the dataset should be directed to corresponding authors.
